# “Bottom-Up” Strategy for the Identification of Novel Soybean Peptides with Angiotensin-Converting Enzyme Inhibitory Activity

**DOI:** 10.1021/acs.jafc.9b07361

**Published:** 2020-01-27

**Authors:** Luca Dellafiora, Raffaele Pugliese, Carlotta Bollati, Fabrizio Gelain, Gianni Galaverna, Anna Arnoldi, Carmen Lammi

**Affiliations:** † Department of Food and Drug, University of Parma, Parma 43124, Italy; ‡ Tissue Engineering Unit, Institute for Stem Cell Biology, Regenerative Medicine and Innovative Therapies-ISBReMIT, Fondazione IRCSS Casa Sollievo della Sofferenza, San Giovanni Rotondo 71013, Foggia, Italy; § Center for Nanomedicine and Tissue Engineering (CNTE), ASST Grande Ospedale Metropolitano Niguarda, Milan 20162, Italy; ∥ Department of Pharmaceutical Sciences, University of Milan, Milan 20133, Italy

**Keywords:** ACE, peptide encapsulation, bioactive peptides, hypotensive peptides, multifunctional peptides, self-assembling peptides

## Abstract

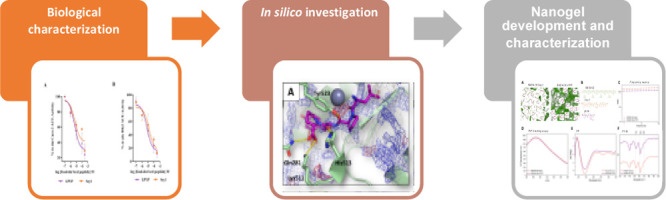

IAVPTGVA (Soy1) and LPYP are two soybean peptides, which display a multifunctional behavior, showing in vitro hypocholesterolemic and hypoglycemic activities. A preliminary screening of their structures using BIOPEP suggested that they might be potential angiotensin-converting enzyme (ACE) inhibitors. Therefore, a bottom-up-aided approach was developed in order to clarify the in vitro hypotensive activity. Soy1 and LPYP dropped the intestinal and renal ACE enzyme activity with IC_50_ values equal to 14.7 ± 0.28 and 5.0 ± 0.28 μM (Caco-2 cells), and 6.0 ± 0.35 and 6.8 ± 0.20 μM (HK-2 cells), respectively. In parallel, a molecular modeling study suggested their capability to act as competitive inhibitors of this enzyme. Finally, in order to increase both their stability and hypotensive properties, a suitable strategy for the harmless control of their release from a nanomaterial was developed through their encapsulation into the RADA16-assembling peptide.

## Introduction

Hypertension is one of the main risk factors for the development of cardiovascular diseases.^1^ A complex interaction of genetic and environmental factors and many other factors (i.e., increased levels of long-term high sodium intake, inadequate dietary intake of potassium and calcium, elevated renin–angiotensin system (RAS) activity, and endothelial dysfunction) is the basis of the pathophysiological development of this disease.^2,3^ In this context, angiotensin-converting enzyme (ACE, EC 3.4.15.1), a dipeptidyl-carboxypeptidase expressed in many tissues (lung, kidney, and intestine), is a key enzyme for blood pressure regulation, being responsible of the conversion of inactive angiotensin I (Ang) into active Ang II, a vasoconstrictive octapeptide that is accountable for hypertension progression.^4^ The inhibition of this enzyme is therefore considered a successful strategy for lowering high blood pressure.

This is also true in the field of hypotensive food peptides: indeed, several peptides from milk, meat, egg, fish, lupin, and soybean sources have been singled out as inhibitors of the ACE activity.^5−8^ Milk proteins have a leading role as a source of ACE inhibitory peptides: in particular, VPP (Val-Pro-Pro) and IPP (Ile- Pro-Pro), two peptides derived from β-casein and κ-casein, are the most active ACE inhibitors from any food source;^9^ their hypotensive effect has been confirmed in vivo in spontaneously hypertensive rats (SHR) fed with sour milk and they are now on the market as ingredients of antihypertensive drinks, such as the Japanese “Calpis” and the Finnish “Evolus”.^10^

IAVPTGVA (Soy1) and LPYP are peptides derived from the hydrolysis of soybean glycinin with pepsin and trypsin,^11^ respectively, which have been demonstrated to be absorbable in Caco2 cell monolayers.^12^ In more detail, recent evidence suggests that both peptides are absorbed by differentiated Caco-2 cells as a function of time and that Soy1 is better absorbed than LPYP after 2 h of incubation in the apical side of monolayers.^12^ Mature enterocytes represent the first physiological barrier that bioactive food peptides encounter after ingestion; therefore, their absorption is a dynamic process which co-exists with their metabolic degradation. In light of this observation, some evidence underlines that during its absorption, Soy1 (IAVPTGVA) is partially metabolized by active Caco-2 cell membrane peptidases in three breakdown fragments (AVPTGVA, IAVP, and IAV), which are also absorbed in the same cellular system.^12^

Both peptides have a multifunctional behavior because in vitro they are either hypocholesterolemic or hypoglycemic.^12−15^ The cholesterol lowering effect is due to the inhibition of 3-hydroxymethylglutaryl coenzyme A reductase (HMGCoAR) and the subsequent activation of the low-density lipoprotein receptor pathway. Moreover, these effects are accompanied by an increase in the phosphorylation level of HMGCoAR on Ser 872 (the inactive form of HMGCoAR), via the activation of the adenosine monophosphate-activated protein kinase (AMPK) pathway.^13^ The capacity to modulate glucose metabolism and uptake is linked to the activation of the AMPK and protein kinase B (Akt) pathways.^14^ The activation of Akt (phosphorylated at Ser 473) leads to the inhibition of glycogen synthase (GS) kinase-3β (GSK3), which in turn regulates the GS activity with a modulation of the hepatic glycogen formation. In parallel, the increased protein levels of glucose transporter type 4 (GLUT4) and glucose transporter type 1 (GLUT1) determine an increased activity of HepG2 cells to clear extracellular glucose. In other experiments, Soy1 and LPYP have been demonstrated to be also capable of inhibiting the activity of dipeptidyl peptidase-IV (DPP-IV), another favorable effect for diabetes prevention.^12,15^

A preliminary screening of the structures of Soy1 and LPYP using BIOPEP (www.uwm.edu.pI/biochemia)^16^ suggested that they might be compatible with a potential behavior as ACE inhibitors. Hence, the first objective of this work was an evaluation of their ACE inhibitory activity. Instead of using the traditional in vitro assay on the enzyme purified from rats or rabbits (mostly used in the literature), our experimentation was based on a cellular assay performed in human intestinal Caco-2 and kidney HK-2 cells, which are among the cells that mostly express this enzyme in the body. In parallel, a molecular modeling study was carried out to investigate their capability to act as competitive inhibitors of ACE, in agreement with previous studies.^17^ The in silico study was based on a structure-based modeling of both ACE domains (namely, the N-domain and C-domain) including pharmacophoric analysis, docking simulations, rescoring procedures, and molecular dynamics.

Finally, because we have previously reported that self-assembling peptide (SAP)-based nanogels are a viable platform for targeting metabolic diseases with bioactive peptides,^18,19^ thanks to their bona fide properties, well-ordered nanostructures, and biocompatibility, here, we provide a smart delivery coating system of Soy1 and LPYP by using a RADA16 hydrogel (Ac-RADARADARADARADA-CONH_2_). The feasibility of this encapsulation strategy was assessed mainly by rheology, thioflavin T (ThT) binding assay, spectroscopy assay [circular dichroism (CD) and attenuated total reflection (ATR)–Fourier transform infrared (FTIR)], and release kinetic experiments.

## Materials and Methods

### Materials

All reagents and solvents were from commercial sources. See the “Supporting Information” for further details on materials and methods.

### In Vitro Digestion of Soy1 and LPYP

Pepsin solution (4 mg/mL in NaCl) was added to Soy1 and LPYP (100 μM) at a 1:100 enzyme-to-substrate ratio (pH 2.0). The digestion was conducted at 37 °C for 90 min under continuous stirring, and then, the pH was adjusted to 7.2 with 1 M NaOH in order to inactivate the enzyme. Then, pancreatin (4 mg/mL in H_2_O) was added at a 1:50 enzyme-to-substrate ratio. After digestion, at 37 °C for 150 min, the enzyme was inactivated by heating at 95 °C for 10 min. Further details regarding the analysis are available in the “Supporting Information”.

### Cell Culture

Caco-2 cells, obtained from the Institut National de la Santé et de la Recherche Médicale (INSERM, Paris), were routinely subcultured as previously described.^18^ HK2 cells from ATCC were cultured using Dulbecco’s modified Eagle’s medium-F12 (DMEM-F12) containing 25 mM glucose, 4 mM stable l-glutamine, 100 U L^–1^ penicillin, and 100 μg L^–1^ streptomycin, supplemented with 10% heat-inactivated fetal bovine serum (FBS Hyclone Laboratories, Logan, UT, USA).

### ACE Activity Cell-Based Assay

Soy1 and LPYP were tested on Caco-2 and HK2 cells (5 × 10^4^/well in black 96-well plates) in 0.1–250.0 μM concentration ranges or vehicle in growth medium for 24 h at 37 °C. For a 2D cell culture on RADA16-Soy1 and RADA-LPYP hydrogels, Caco-2 cells were seeded on the surface of the abovementioned hydrogels at a density of 5 × 10^4^/well. On the next day, the ACE inhibitory activity was measured using an ACE1 Activity Assay Kit (BioVision, Milpitas Blvd., Milpitas, CA, USA) following the manufacturer’s protocol. See the “Supporting Information” for further details on Acer activity cell-based assay.

### In Silico Modeling

A molecular modeling approach was used to investigate the interaction of peptides with the N- and C-domain of human ACE from a molecular perspective. In more detail, the computational analysis relied on pharmacophoric modeling followed by docking simulations coupled to rescoring procedures to assess the capability of peptides to fit the catalytic sites of both domains, as previously reported.^17^ Then, a 50 ns dynamic simulation study was applied to assess their capability to persist therein. See the “Supporting Information” for further details on molecular modeling.

### Synthesis and Purification of RADA16

As we previously reported,^18^ the RADA16 molecule was synthesized by fluorenylmethoxycarbonyl solid-phase peptide synthesis and purified by high-performance liquid chromatography. The purity of the lyophilized peptide was tested by single quadrupole mass spectrometry using an Alliance-3100 LC–MS instrument. After lyophilization, RADA16 was dissolved at 1% (w/v) in distilled waters.

### Rheological Measurement

Rheological measurement was performed using an AR-2000ex Rheometer (TA Instruments, New Castle, DE, USA) with a 20 mm acrylic truncated plate. All peptide samples were tested at the concentration of 1% (w/v), and the sample stage was set to 25 °C. The storage modulus was recorded as a function of angular frequency (0.1–100 Hz) at a fixed strain of 1%.

### ThT Spectroscopy Assay

The propensity of assembled peptides to form cross-β fibril structures was studied using ThT binding assay, as previously described.^18^

### CD Spectroscopy Assay

CD spectra of peptide samples were recorded in the continuous scanning mode (190–300 nm) at 25 °C using Jasco J-810 (JASCO Corp., Tokyo, Japan) spectropolarimeter. All spectra were collected using a 1 mm path length quartz cell and averaged over three accumulations (speed: 10 nm min^–1^). A reference spectrum of distilled water was recorded and subtracted from each spectrum. The estimation of the peptide secondary structure was achieved using a literature method.^20^

### FTIR Spectroscopy Analysis

Similar to our previous report,^18^ FT-IR analysis was performed on peptides dissolved at a final concentration of 1% (w/v) in distilled water. More details are available in the “Supporting Information”.

### Kinetics of Soy1 and LPYP Peptide Release from the Nanogels

The peptide leaking from the nanogels as a function of time was measured dissolving the nanogels in phosphate-buffered saline (PBS) and measuring the concentrations of released peptides after 60, 180, and 360 min of incubation using a method previously described.^19^ See the “Supporting Information” for further details on kinetic evaluation of both peptides’ release.

### Cell Viability Test

Caco-2 cells were seeded on the surface of RADA16-Soy1 and RADA-LPYP hydrogels at a density of 5 × 10^4^/well and cultured for 6 days. Intestinal cell growth was qualitatively evaluated by collecting images using a Zeiss Axioplan 2 microscope (Oberkochen, Germany). Finally, MTT experiments were carried out using a method previously reported.^21^

### Statistical Analysis

Statistical analyses were carried out by one-way ANOVA using GraphPad Prism 6 (GraphPad, La Jolla, CA, USA). The values were expressed as mean ± s.d. of three independent experiments; each experiment was performed in triplicate; and *p* values < 0.05 were considered to be significant.

## Results and Discussion

### Soy1 and LPYP Inhibit the In Situ ACE Activity on Human Intestinal Caco-2 and Kidney HK-2 Cells

**Figure 1:**
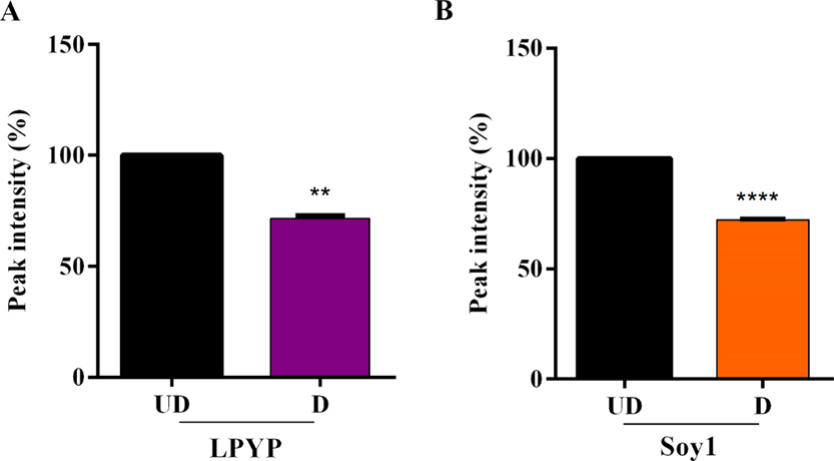
In vitro gastrointestinal digestion. LPYP (A) and Soy1 (B) were codigested with pepsin for 90 min and pancreatin for 150 min. After digestion, LPYP and Soy1 (D) were degraded by only 28.5 ± 1.4 and 27.7 ± 0.3%, respectively, vs undigested peptide. Data represent the mean ± s.d. of three independent experiments performed in triplicate.

Their metabolic propensity to be degraded by peptidases, which are physiologically active along the entire gastrointestinal tract, might dramatically influence the bioactivity of food peptides. The literature provides many studies dealing with the assessment of food bioactive peptide stability to the simulated gastric digestion.^22,23^ In order to in-depth characterize the multifunctional behavior of Soy1 and LPYP, their stability toward the in vitro gastric digestion was assessed using pepsin and pancreatin. Figure 1 indicates that after codigestion with these enzymes, LPYP and Soy1 are degraded by only 28.5 ± 1.4 and 27.7 ± 0.3%, respectively. These results highlight that both Soy1 and LPYP are noteworthy stable to the in vitro gastric digestion.

**Figure 2:**
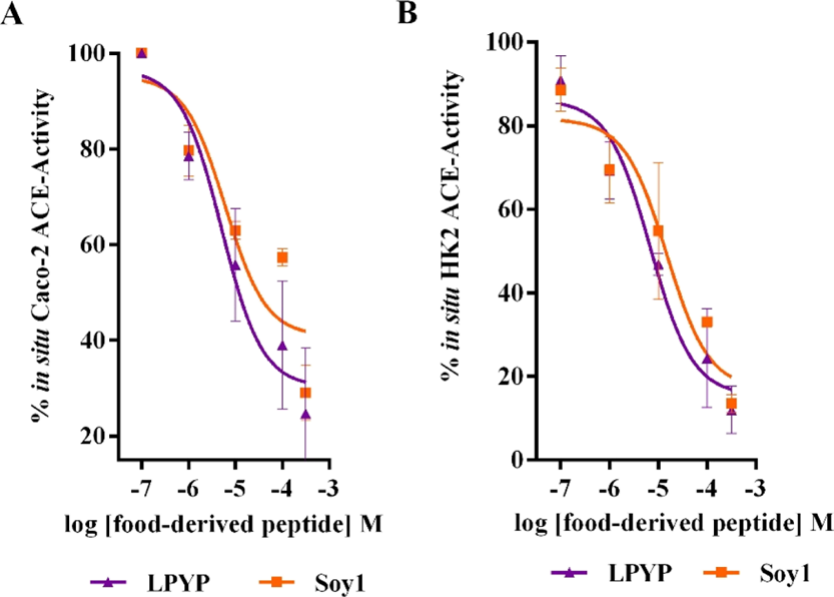
In situ evaluation of the ACE activity. Soy1 and LPYP reduce in situ the ACE activity with a dose-response trend (A) in nondifferentiated human Caco-2 cells (IC_50_ values equal to 14.7 ± 0.28 and 5.0 ± 0.28 μM, respectively) and (B) in renal HK-2 cells (IC_50_ values equal to 6.0 ± 0.35 and 6.8 ± 0.20 μM, respectively). Data represent the mean ± s.d. of three independent experiments performed in triplicate.

Based on these results, in order to investigate the potential hypotensive effect of Soy1 and LPYP, their ability to drop in situ the ACE activity was evaluated using a cell-based assay. In particular, Caco-2 and HK-2 cells (5 × 10^4^/well) were treated with Soy1 and LPYP (0.1–250 μM) overnight. The following day, cells were lysated and the ACE activity was measured directly in the cell lysates using a fluorescent ACE substrate; in this assay, the fluorescent signal is proportional to the enzyme activity. As shown in Figure 2, Soy1 and LPYP reduced the enzyme activity with a dose-response trend in both biological systems (Caco-2 and HK-2 cells). In particular, Soy1 and LPYP displayed calculated IC_50_ values equal to 14.7 ± 0.28 and 5.0 ± 0.28 μM in Caco-2 cells, respectively (Figure 2A), whereas the same peptides showed IC_50_ values equal to 6.0 ± 0.35 and 6.8 ± 0.20 μM in HK-2 cells, respectively (Figure 2B).

The literature provides many examples of studies in which different food-derived peptides target the in vitro activity of ACE. In all these studies, their biochemical characterization has been carried out using in vitro tests employing the purified recombinant ACE enzymes from different animal species, such as pigs and rabbits. Although the ACE sequence is highly conserved among species,^24^ the only use of biochemical tools involving the purified ACE enzymes and a standard substrate provide only insufficient characterization of the activity before performing expensive in vivo experimental studies. On the contrary, a cell-based assay is certainly more helpful because it allows the investigation of the enzyme in its natural environment and to account for possible metabolic modifications of the peptide structure and activity. For this reason, two cellular systems, human intestinal Caco-2 and renal HK-2 cells, were chosen to characterize the potential inhibitory activity of Soy1 and LPYP in a more realistic way. In particular, the intestine is the first physiological barrier that peptides from food sources encounter after ingestion and it is well-known that the intestine and kidney express a high level of ACE, where the ACE and RAS systems play a key role in blood pressure regulation.^25,26^ Our findings clearly suggest that both soybean peptides show an outstanding ACE inhibitory activity: LPYP displays comparable IC_50_ values in Caco-2 and HK-2 cells, whereas Soy1 is twofold more active at the renal level than at the intestinal level. This may be explained by the propensity of Soy1 to undergo a metabolic degradation by active peptidases that are expressed in the apical side of intestinal cells. Indeed, a recent study has demonstrated that intestinal cells absorb both LPYP and Soy1, but during this process, the latter is partially cleaved into shorter peptides (AVPTGVA, IAVP, and IAV).^12^

It is well-recognized that soybean proteins contain many bioactive peptides exerting multiple health benefits, that is, hypocholesterolemic, antidiabetic, antitumor, and hypotensive activity. In this context, LAIPVNKP and LPHF are two ACE inhibitors reported in the literature with IC_50_ values of 70 and 670 μM, respectively.^27^ Moreover, after the hydrolysis of soybean proteins with pepsin, five peptides have been identified showing in vitro and in vivo hypotensive activity. In particular, IA, YLAGNQ, FFL, IYLL, and VMDKPQG show IC_50_ values of 153, 14, 37, 42, and 39 μM, respectively. Their blood pressure-lowering activity has been also confirmed in vivo on SHR models.^28^ Moreover, peptides SPYP and WL, obtained from the hydrolysis of soybean glycinin by acid proteinase from *Monascus purpureus*, have been shown to be able to inhibit the ACE activity in vitro with IC_50_ values equal to 850 and 65 μM, respectively.^27^ Surprisingly, among the known ACE inhibitory peptides from soybean proteins, SPYP and LPYP are very similar, the only difference relaying on a single amino acid residue. LPYP is 170-fold more potent than SPYP, suggesting that the presence of a hydrophobic amino acid residue with an aliphatic chain (Leu) instead of a polar residue with a hydroxymethyl group (Ser) leads to an impressive potency gain.

Because the literature on ACE inhibitory peptides from food is very extensive, a correlation between their physicochemical and structural properties with bioactivity is well-established. In particular, to induce ACE inhibition, hydrophobic peptides (2–8 amino acid residues) should be present in the N-terminal hydrophobic amino acids, especially those with aliphatic chains such as Gly, Ile, Leu, and Val, and at the C-terminal amino acids with cyclic or aromatic rings (Pro, Tyr, and Trp).^29,30^ Many peptides derived from food proteins contain Pro at the C-terminal, a rule concerning mostly short peptides.^31^ The ACE inhibitory activity is furthermore improved by the simultaneous occurrence of a C-terminal Pro and an N-terminal branched-side aliphatic amino acid. Indeed, our results are in agreement with these structure–activity relationships. In light of all these observations and in order to gain an insight of the binding mode of both peptides with the ACE, in silico investigation was performed.

### Molecular Modeling Studies

Soy1 and LPYP underwent a molecular modeling study in order to investigate their possible interaction with the N- and C-domain of human ACE at the molecular level. The in silico study consisted in the pharmacophoric description of both the catalytic sites of ACE, followed by docking simulations coupled to rescoring procedures to better evaluate the protein–peptide interaction, in agreement with previous studies.^32,33^ The top-scored docking pose in each domain was then compared with the respective pharmacophoric fingerprint, providing a qualitative structure–activity relationship analysis. Finally, LPYP, which showed the best IC_50_ value and the highest computational scores (vide infra), underwent 50 ns molecular dynamic simulations to study the geometrical stability of its interaction over time.

As previously reported, the two catalytic sites showed a largely conserved sequence identity and a similar spatial organization of residues, determining a comparable pocket shape and a similar distribution of pharmacophoric properties.^17^ Concerning the results of docking simulations and rescoring procedures, LPYP but not Soy1 seemed able to favorably interact with the two catalytic sites of ACE. Indeed, on the one side, LPYP recorded a HINT score of 2570 and 2830 within the N- and C-domain, respectively. On the other side, Soy1 recorded a HINT score of 20 and −374 within the N- and C-domain, respectively. Notably, the HINT score relates to the free energy of binding and, specifically, the higher the score the stronger the interaction. Conversely, negative scores, as well as scores proximal to zero, may indicate the lack of appreciable interaction, as previously reported.^32,34,35^ On this basis, the capability of Soy1 to interact with the two catalytic sites wasdetermined less favorable than the one of LPYP.

**Figure 3:**
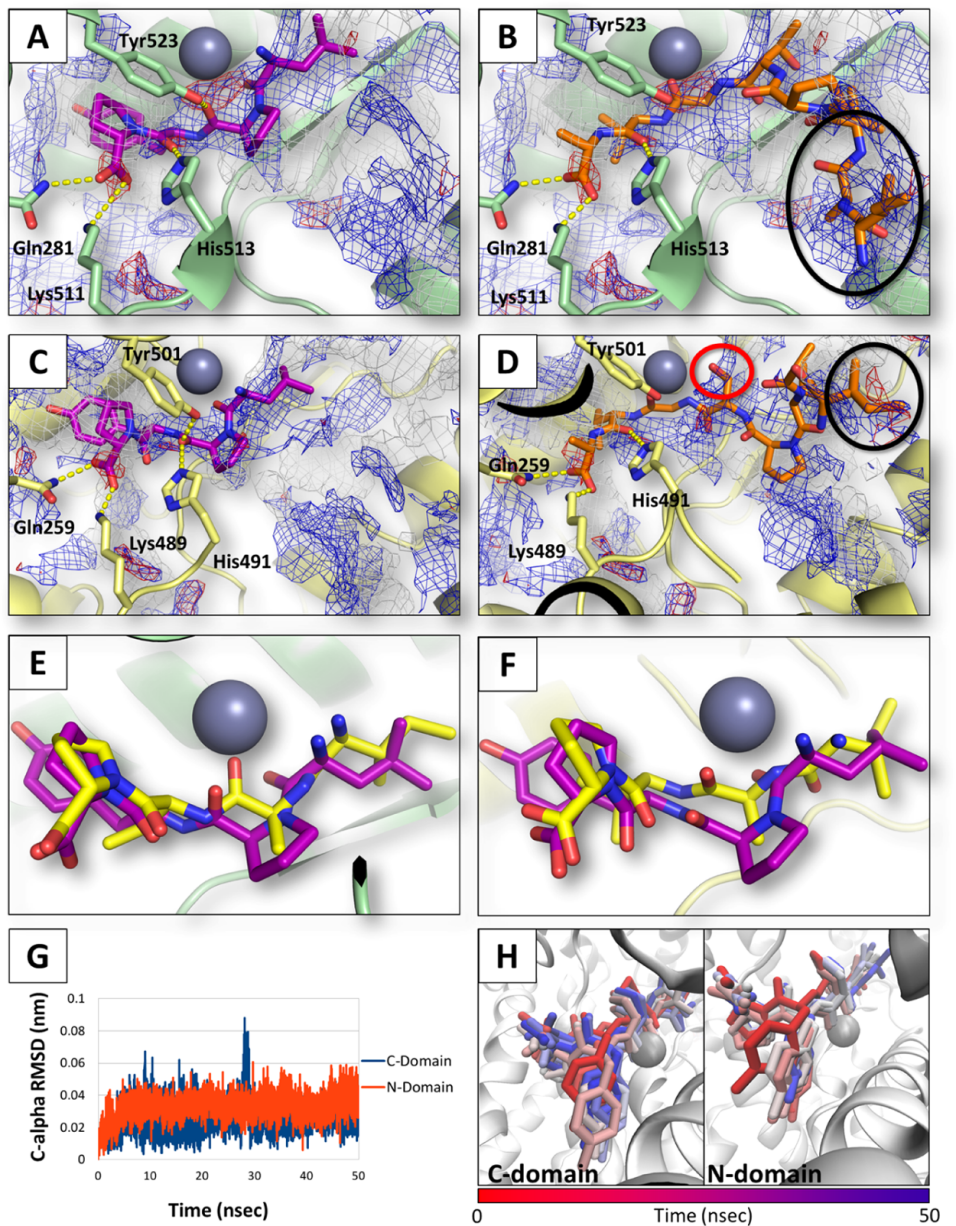
Molecular modeling results. The protein is represented in cartoon, while peptides and residues involved in polar interactions are represented in sticks. Spheres represent Zn ions. Gray, red, and blue meshes indicate regions sterically and energetically favorable to receive hydrophobic, hydrogen bond acceptor, and hydrogen bond donor groups, respectively. Polar interactions are indicated by yellow-dotted lines. The red and black circles indicate hydrophobic-polar and acid–acid interferences. (A) LPYP within the C-domain. (B) Soy1 within the C-domain. (C) LPYP within the N-domain. (D) Soy1 within the N-domain. (E) Superimposition of IAVP (yellow) to LPYP (purple) within the C-domain. (F) Superimposition of IAVP (yellow) to LPYP (purple) within the N-domain. (G) rmsd plots of LPYP within the catalytic site of the C- and N-domain of ACE. (H) Time-step representation of LPYP trajectories within the N- and C-domain of ACE. The from-red-to-blue color switch indicates the stepwise changes of ligand coordinates over time (50 ns).

The docking analysis poses of LPYP in each ACE catalytic site with respect to their respective pharmacophoric fingerprints provided a molecular rationale to such results. As shown in Figure 3, LPYP engaged both sites via a multiple hydrogen bond network starkly complying with the pharmacophoric fingerprint of the two pockets. Conversely, the N-terminal residues of Soy1 were found not fully matching neither of the two catalytic sites, although the peptide could form hydrogen bonds with its C-terminal residue. Specifically, both hydrophobic-polar and acid–acid interferences were found, as shown in Figure 3B,D. This evidence provided a structural rationale explaining the diverse scores recorded by LPYP and Soy1, pointing out the better capability of the former to interact with the catalytic sites of ACE. The low compliance of Soy1 to the catalytic sites of ACE eventually suggested its presumably low capability to inhibit ACE via a competitive mechanism with the catalytic site. Nonetheless, this result was in apparent contrast with the experimental evidence reported above stating its ACE inhibitory activity, although of a lower intensity than LPYP. However, ACE inhibitory peptides may also interact in regions other than the catalytic sites changing the capability of substrates to reach the catalytic core.^36^ Therefore, Soy1 might act through mechanisms that do not require competitive binding at the catalytic sites.

Considering that Soy1 may be hydrolyzed by cells releasing fragments such as AVPTGVA, IAVPT, and IAVP,^12^ these fragments were also submitted to the docking and rescoring procedure to assess their possible capability to fit the catalytic sites of ACE. AVPTGVA, IAVPT, and IAVP recorded 52, 50, and 1956 HINT scores within the N-domain, respectively. Conversely, they recorded 793, 624, and 2164 HINT scores within the C-domain, respectively. In particular, IAVP markedly complied the pharmacophoric requirements of both pockets strongly retracing the mode of binding of LPYP (Figure 3). Moreover, on the basis of the obtained scores, AVPTGVA and IAVPT were found to better satisfy the pocket requirements of the C-domain than those of the N-domain (wherein their interaction was considered unfavorable because of the low scores recorded). These results might point to their possible preferential interaction and inhibition with the N-domain. This feature might deserve future investigations in order to identify domain-specific inhibitory peptides.

Overall, these results support the full compliance of LPYP as an ACE inhibitor via competitive mechanisms at the catalytic sites of ACE. Conversely, the capability of Soy1 to interact with the catalytic sites was determined less-favored than that of LPYP. However, other noncompetitive mechanisms could not be excluded. In addition, Soy1 fragments released by cell peptidases might competitively inhibit ACE concurring to the overall inhibitory potential of Soy1.

Finally, LPYP, which recorded both the best IC_50_ in experimental trials and the highest scores in computational analysis, was submitted to molecular dynamic simulations (50 ns) to check the capability to persist within the catalytic sites of ACE over time. The interaction of LPYP was found geometrically stable in both ACE domains, as shown by the low root-mean-square deviation (rmsd) fluctuations of C-α (Figure 3G). In addition, the inspection of LPYP trajectories revealed its persistence and stability within both the catalytic sites (Figure 3H), further supporting its capability to inhibit ACE via competitive mechanisms.

### Supramolecular Approach for the Development of Soy1- and LPYP-Based Nanogels: Mechanical, Structural, and Biological Characterizations

**Figure 4:**
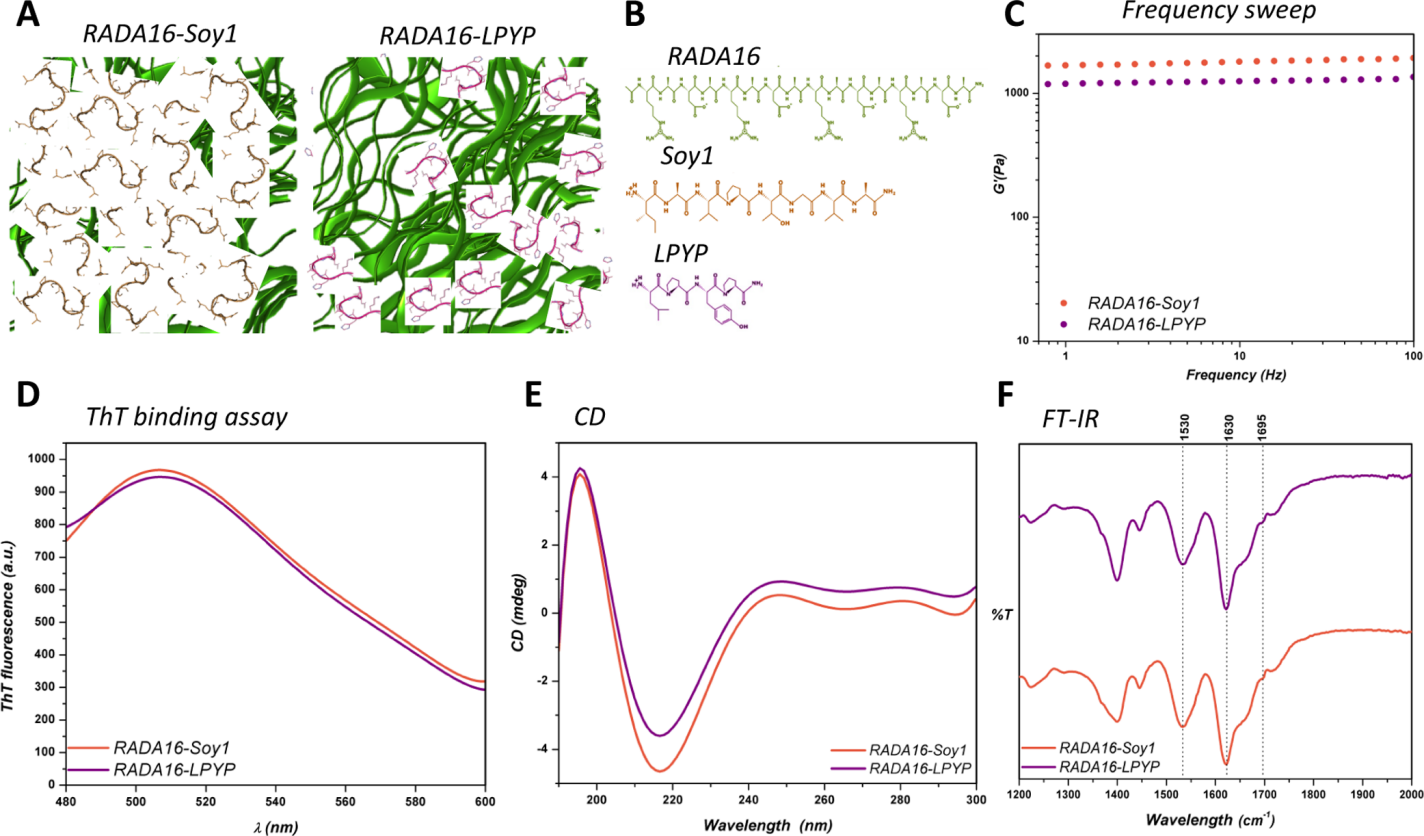
Characterization of SAP nanogels. (A) Cartoon representation and (B) chemical structures of RADA16, RADA16-Soy1, and RADA16-LPYP nanogels. (C) Biomechanical characterization of RADA16-Soy1 and RADA16-LPYP nanogels via a frequency sweep test (0.1–100 Hz, 1% strain). (D) ThT emission spectra of RADA16-Soy1 and RADA16-LPYP nanogels: their affinity for ThT may be ascribable to the presence of cross-β fibril structures. (E) CD spectrum of RADA16-Soy1 and RADA16-LPYP in solution showing the presence of β-sheet assemblies. (F) FTIR analysis of RADA16-Soy and RADA16-LPYP with peaks at ∼1630 and ∼1695 cm^–1^ (amide I region) and 1530 cm^–1^ (amide II region) typically associated with β-sheet signatures.

SAPs are a promising class of supramolecular nanomaterials for controlled drug delivery applications and beyond. Here, we report the feasibility of encapsulating the bioactive peptides Soy1 and LPYP (Figure 4A,B) into the self-assembly peptide RADA16.^37^

In order to assess the ability of RADA16 to support the slow release of both Soy1 and LPYP peptides, 1% (w/v) of RADA16-Soy1 or RADA16-LPYP nanogels was prepared to characterize their viscoelastic properties. Rheological measurements were performed to estimate the elastic response (*G*′) of nanogels, by varying frequencies of applied oscillatory stress at constant strain (0.1–100 Hz, 1% strain). All pre-assembled solutions showed typical soft hydrogel profiles,^19^ featuring a *G*′ modulus of ∼1800 and ∼1100 Pa for RADA16-Soy and RADA16-LPYP, respectively (Figure 4C).

The amyloidogenic nature of the nanogels was studied using a ThT binding assay (Figure 4D). This assay enables evaluation of the amyloidogenic structures and cross-β fibril formation of materials because β-rich structures feature ThT-binding sites. ThT assay resulted in high fluorescence levels, as well as a typical amyloid-binding emission signal (peak at ∼490 nm), thus establishing the β-rich amyloidogenic nature of both nanogels. To study the secondary structure of the nanogels in solution, CD spectroscopy was carried out. As expected, both nanogels exhibited a CD signal comprising a negative peak near 215 nm and a positive peak at ∼195 nm characteristic of a β-sheet conformation (Figure 4E). To gain further information about the nanogel secondary structure, a literature method has been used,^20^ which suggested that RADA16-Soy1 has 84% of β-sheet structures, whereas RADA16-LPYP has 78%. Thus, the CD spectra are in accordance with ThT binding assay. Furthermore, the β-sheet structural arrangements of RADA16-Soy and RADA16-LPYP were also supported by ATR–FTIR spectroscopy (Figure 4F), which displayed two peaks at ∼1630 and ∼1695 cm^–1^ (amide I region) and one peak centered around 1530 cm^–1^ (amide II region) typically associated with β-sheet signatures.

**Figure 5:**
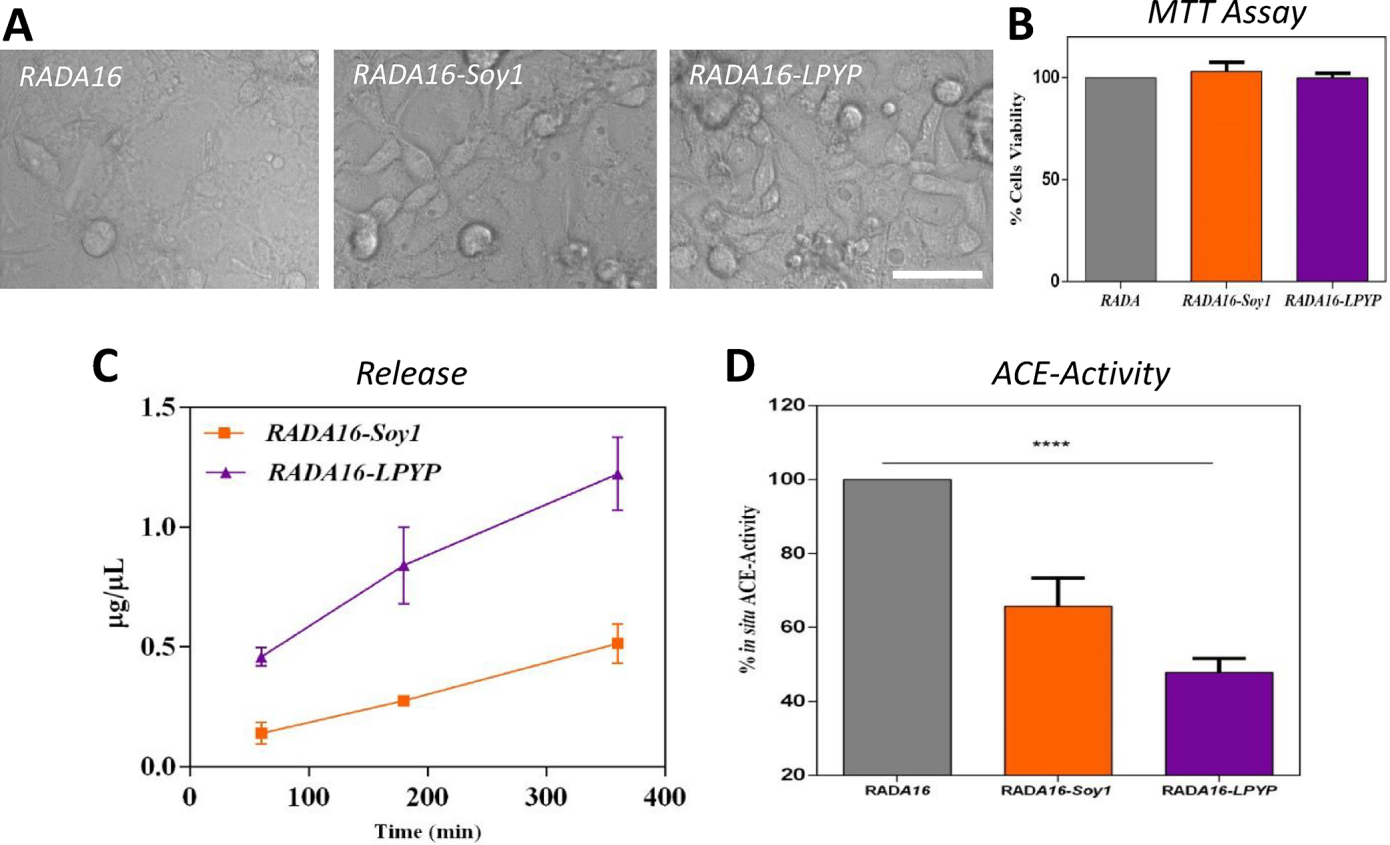
Biological characterization of the nanogels. Photographs of the Caco-2 cells grown on top of RADA, RADA-Soy1, and RADA-LPYP hydrogels for 6 days (A); cell viability tests performed by MTT assay (B); kinetics of peptide release as a function of time (C); and evaluation of the in situ ACE inhibitory effects on human intestinal Caco-2 cells (D). Data represent the mean ± s.d. of three independent experiments performed in triplicate; ****p < 0.0001.

To investigate the capability of Soy1- and LPYP-based nanogels to modulate the ACE activity, in situ experiments were carried out on human intestinal Caco-2 cells (Figure 5A–D). Briefly, a total of 5 × 10^4^/well Caco-2 cells were seeded directly on top of the coating nanogels in which Soy1 and LPYP peptides had been entangled at the concentration of 1.0 μM. Cells were cultured for 6 days in order to evaluate the ability of both soybean peptide-based nanogels to act as cell culture coating. As shown in Figure 5A, Caco-2 cells were able to grow on top of both coating nanogels without significant morphological variation compared to Caco-2 cells, which grew on top of the RADA16 hydrogel alone. Indeed, as MTT results clearly suggested, no cytotoxicity effects were observed even after 6 days of cell culture (Figure 5B). After 6 days, Caco-2 cells are in a proliferative stage, they reach confluence and even though they are not fully differentiated in mature enterocytes, they express enough amounts of active membrane peptidases, that is, DPP-IV and ACE.^38^ For this reason, this cellular system, which has already been used to monitor the in situ activity of DPP-IV, can be also utilized to evaluate the ACE activity.^39,40^ Based on these results, the kinetic of each peptide release was assessed using a literature method, which is based on chelating the peptide bonds by Cu(II) in alkaline media and monitoring the change of absorbance at 330 nm.^41^ Using this method, it was demonstrated that both peptides are released by the coating hydrogel as a function of time with a different behavior. In detail, released Soy1 concentrations are 0.13 ± 0.04, 0.27 ± 0.003, and 0.51 ± 0.08 μg μL^–1^, whereas released LPYP peptide concentrations are 0.46 ± 0.04, 0.84 ± 0.15, and 1.22 ± 0.16 μg μL^–1^, respectively, after 60, 180, and 360 min of incubation in PBS (Figure 5C). The LPYP peptide is released faster than Soy1 probably because it is less hydrophobic (LPYP hydrophobicity is equal to 6.22 kcal mol^–1^ and that of Soy1 is equal to 8.40 kcal mol^–1^). This explains why LPYP may more easily leak from the entangled nanofibrous domains of the hydrogels than Soy1.

Furthermore, the ability of both coating nanogels to inhibit the ACE activity was evaluated in situ on human intestinal Caco-2 cells. Findings clearly underline that both RADA16-Soy1 and RADA16-LPYP maintain their ability to reduce the enzyme activity by 40 and 60%, respectively (Figure 5D). In detail, when Soy1 and LPYP peptides are entrapped in the coating nanogels at the concentration of 1 μM, they drop the ACE activity by 34.3 ± 7.6 and 52.2 ± 3.8%, respectively, suggesting a clear improvement of their inhibitory activity. These results are in agreement with the relative activity (data not shown) of each peptide, that is, LPYP is more active than Soy1. Overall, these results clearly support our hypothesis of developing a suitable smart delivery coating strategy for the harmless control of ACE inhibitory peptides as a new approach for improving their activity and stability.

## Supporting Information Available

More detailed description of the materials and methods section (PDF)

## Abbreviations

ACE: angiotensin-converting enzyme
Akt: protein kinase B
AMPK: adenosine monophosphate-activated protein kinase
CD: circular dichroism
DMEM: Dulbecco’s modified Eagle’s medium
DPP-IV: dipeptidyl peptidase-IV
FBS: fetal bovine serum
FT-IR: Fourier transform infrared spectroscopy
GLUT1: glucose transporter type 1
GLUT4: glucose transporter type 4
GS: glycogen synthase
GSK3: glycogen synthase kinase-3β
HMGCoAR: 3-hydroxymethylglutaryl coenzyme A reductase
PBS: phosphate-buffered saline
RAS: renin–angiotensin system
SAPs: self-assembling peptides
SHRs: spontaneous hypertensive rats
ThT: thioflavin T
