# Supplementary material for: “Bottom-Up” Strategy for the Identification of Novel Soybean Peptides with Angiotensin-Converting Enzyme Inhibitory Activity
Source: J Agric Food Chem. 2020 Jan 27;68(7):2082–90. doi: 10.1021/acs.jafc.9b07361 (PMC7997397; doi:10.1021/acs.jafc.9b07361)
Supplement: Supplementary file 1 — jf9b07361_si_001.pdf [file jf9b07361_si_001.pdf]

“Bottom up” strategy for the identification of novel soybean peptides with in vitro hypotensive activity targeting ACE enzyme

Luca Dellafiora[1], Raffaele Pugliese[2], Carlotta Bollati[4], Fabrizio Gelain[2,3], Gianni Galaverna[1], Anna Arnoldi[4], Carmen Lammi[4*]

[1] Department of Food and Drug, University of Parma, Parma, Italy.

[2] Tissue Engineering Unit, Institute for Stem Cell Biology, Regenerative Medicine and Innovative Therapies-ISBReMIT, Fondazione IRCSS Casa Sollievo della Sofferenza, San Giovanni Rotondo (FG), Italy

[3] Center for Nanomedicine and Tissue Engineering (CNTE), ASST Grande Ospedale Metropolitano Niguarda, Milan, Italy

[4] Department of Pharmaceutical Sciences, University of Milan, Milan, Italy

*Corresponding author: Phone: +39-0250319912; FAX: +39-0250319359; Email: carmen.lammi@unimi.it.

Materials and Methods

Materials. Fmoc-Arg(Pbf)-OH, Fmoc-Ala-OH, and Fmoc-Asp(OtBu)-OH were purchased from the Aapptec (Louisville, USA) and used as received. N,N-dimethylformamide (DMF), N,N-diisopropylethylamine (DIPEA), N-methyl-2-pyrrolidone (NMP), trifluoroacetic acid (TFA) and triisopropylsilane (TIS) were purchased from VWR (Radnor, USA). N,N,N’,N’-tetramethyl-O-(1H-benzotriazol-1-yl)uronium hexafluorophosphate (HBTU), 1-hydroxybenzotriazole hydrate (HoBT), 4-metylpiperidin and Thioflavin T were purchased from Sigma-Aldrich. HPLC grade water (resistivity 18 MΩ cm) and DPBS (pH 7.4) were purchased from Thermo Fisher scientific (Waltham, USA).

***In vitro* digestion of Soy1 and LPYP.** Pepsin solution (4 mg/mL in NaCl) was added to Soy1 and LPYP (100 μM) at a 1:100 enzyme to substrate ratio (pH 2.0). The digestion was conducted at 37 °C for 90 min under continuous stirring, and then the pH was adjusted to 7.2 with 1 M NaOH in order to inactivate the enzyme. Then, pancreatin (4 mg/mL in H_2O) was added at a 1:50 enzyme to substrate ratio. After digestion at 37 °C for 150 min, the enzyme was inactivated by heating at 95 °C for 10 min. Centrifugal filter devices (cutoff 3 kDa) from Amicon Bioseparations (Millipore Corporation, Bedford, MA, USA) were used to remove traces of the employed enzymes. Soy1 and LPYP samples undigested and after digestion were analyzed on a SL IT mass spectrometer interfaced with a HPLC- Chip Cube source (Agilent Technologies, Palo Alto, CA, USA). Data were processed with MSD Trap control 4.2, and Data analysis 4.2 version (Agilent Technologies). The chromatographic separation was performed using a 1200 HPLC system equipped with a binary pump. The peptide enrichment was performed on a 160 nL enrichment column (Zorbax 300SB-C18, 5 μm pore size), followed by separation on a 150 mm × 75 μm analytical column packed (Zorbax300SB-C18, 5 μm pore size). The nanopump gradient program were as follows: Soy1 (IAVPTGVA) analysis, 3% solvent B (0 min), 60% solvent B (0–6 min), and back to 5% in 2 min, and LPYP analysis, 5% solvent B (0 min), 80% solvent B (0–5 min), and back to 5% in 1 min. A post acquisition time of 5 minutes at the initial chromatographic conditions was used after each run. Data acquisition was accomplished in positive ionization mode. Capillary voltage was −1,950 V, with endplate offset −500 V, skimmer -40 V, drying gas flow 5 L/min, drying gas temperature 300 °C, nebulizer gas pressure 18 psi, trap drive 39.9, scan range m/z 150–2000, averages of five spectra, ion charge control (ICC) target 30,000. The targeted assay was performed by targeting the precursor ions at *m/z* 727.2 for Soy1 and *m/z* 489.3 for LPYP, respectively. A blank was analyzed between samples to ensure

absence of carryover effects. The peptides were identified by comparing their retention times, MS profiles, and MS/MS fragmentation spectra with those of authentic standards.

ACE activity cell-based assay. Soy1 and LPYP were tested on Caco-2 and HK2 cells (5×10^4/well in black 96-well plates) at 0.1-250.0 μM concentration ranges or vehicle in growth medium for 24 h at 37 °C. For 2D cell culture on RADA16-Soy1 and RADA-LPYP hydrogels, Caco-2 cells were seeded on the surface of the above-mentioned hydrogels at the density of 5×10^4/well. The next day, The ACE inhibitory activity was measured using the ACE1 Activity Assay Kit (Biovision, Milpitas Blvd., Milpitias, CA, USA) following the manufacture's protocol. Briefly, cells were scraped in 30.0 μL of ice-cold ACE lysis buffer and transferred in an ice-cold microcentrifuge tube. After centrifugation at 13,300 g for 15 min at 4 °C, the supernatant was recovered and transferred into a new ice-cold tube. Total proteins were quantified by Bradford method, and 2.0 μg of proteins (the equivalent of 2 μL) were added to 18.0 μL of ACE lysis buffer in each well in a black 96-well plates with clear bottoms. For the background control, 20.0 μL of ACE lysis buffer were added to 20.0 μL of ACE assay buffer. Then, 20.0 μL of 4% of ACE substrate (in assay buffer) was added in each well except the background one and the fluorescence (Ex/Em 330/430 nm) was measured in a kinetic mode for 10 min at 37 °C using Synergy H1 plate reader.

Molecular Modeling

The molecular modeling study aimed at describing the interaction between peptides and both the N- and C-domain of human ACE. The study relied on pharmacophore modeling, docking studies

and molecular dynamic simulations, as detailed below.

Model Preparation. The models for the C- and N-domain of human ACE were derived from the structures recorded into the Protein Data Bank (http://www.rcsb.org) with PDB codes 4APH and 4BZS, respectively.[1]

Protein structures were processed using the software Sybyl, version 8.1 (www.certara.com), as previously reported. [2] Briefly, all atoms of both structures were checked for atom- and bond-type assignments and amino- and carboxyl-terminal groups were set as protonated and deprotonated, respectively. Hydrogen atoms were computationally added to the protein and energetically minimized using the Powell algorithm (the coverage gradient was set ≤0.5 kcal/(mol Å) with a maximum of 1500 cycles). All sets of small molecules, but not the Zn ions, co-crystallized within the catalytic sites were removed to prepare the model for docking simulations.

Peptides were designed using the "Build Protein" tool of the "Biopolymer" module of Sybyl, version 8.1 (www.certara.com). Then, they were energetically minimized using the Powell algorithm with a coverage gradient ≤ 0.05 kcal/(mol Å) with a maximum of 500 cycles.

Pharmacophoric Modeling. The pharmacophoric modeling aimed at describing the physicochemical properties of catalytic sites in terms of distribution of hydrophobic and hydrophilic features. The binding site of both domains of ACE was defined using the Flapsite tool of the FLAP software while the GRID algorithm was used to investigate the corresponding pharmacophoric space [3]. In particular, the DRY probe was used to describe potential hydrophobic interactions, while the sp2 carbonyl oxygen (O) and the neutral flat amino (N1) probes were used to describe the hydrogen bond acceptor and donor capacity of the target,

respectively.

Docking Study and Rescoring Procedure. The docking study aimed at investigating the architectures of binding of peptides within the catalytic sites of ACE domains. The GOLD software (version 5.7)[4] was used to perform all of the docking simulations, while a rescoring procedure using the HINT scoring function[5] was performed for the better evaluation of the protein-peptides interaction. In particular, HINT score relates to the free energy of binding (the higher the score means the stronger the interaction, while negative scores indicate the lack of appreciable interaction)[6] Notably, the coupling of docking simulations using GOLD and rescoring procedures using HINT already succeed in identifying enzymes inhibitor, as previously showed.[6, 7] Software setting and docking protocol were used as reported previously.[8] Briefly, the explorable space available for docking peptides was set 10 Å around the Zn ion. In addition, the interaction of C-terminal carboxylic group of peptides was restrained in agreement with the arrangement of carboxylic group of captopril, as reported by crystallographic study,[9] to speed up the spatial search.

GOLD uses a Lamarckian genetic algorithm, and scores may slightly change from run to run. Therefore, to exclude a non-causative score assignment, simulations were run in quintuplicate, and the mean values are reported, in agreement with previous studies.[8]

Molecular Dynamic Simulations. Molecular dynamic (MD) simulations were performed to study the dynamic of interactions between peptides and the ACE domains over time. The best scored binding poses calculated by docking simulations were used as input for MD. MD simulations were performed using GROMACS (version 5.1.4) with CHARMM27 all-atom force field

parameters support [REF], in agreement with a previous study.[10] Briefly, protein-peptides complexes were solvated with SPCE waters in a cubic periodic boundary condition, and counter ions (Na^+ and Cl^-) were added to neutralize the system. Prior to MD simulation the systems were energetically minimized to avoid steric clashes and to correct improper geometries using the steepest descent algorithm with a maximum of 5000 steps. Afterwards, all the systems underwent isothermal (300 K, coupling time 2psec) and isobaric (1 bar, coupling time 2 psec) 100 psec simulations before running 50 nsec simulations (300 K with a coupling time of 0.1 psec and 1 bar with a coupling time of 2.0 psec).

Fourier transform infrared spectroscopy (FT-IR) analysis. Similar to our previous report,[18] FT-IR analysis was performed on peptides dissolved at a final concentration of 1% (w/v) in distilled water. All spectra were collected in attenuated total reflection (ATR) using Perkin Elmer Spectrum 100 spectrometer. Twenty acquisitions were recorded for each spectrum, using the following condition: 4 cm^{-1} spectrum resolution, 25 kHz scan speed, 1000 scan co-addition and a triangular apodization.

Kinetic of Soy1 and LPYP peptide release from the nanogels. The peptide leaking from the nanogels as a function of time was measured dissolving the nanogels in PBS and measuring the concentrations of released peptides after 60, 180, and 360 min of incubation by using a method previously described. Briefly, a sterile solution of peptone from casein at 10 mg mL^{-1} in water was prepared and used as standard for the calibration curves. Reaction mixtures containing 9.5 µL of the solutions of released Soy1 and LPYP or peptone solution, 90.5 µL water, 95 µL NaOH 6% (w/w) in water, and 9.5 µL of active reagent (containing 0.6 M sodium citrate, 0.9 M sodium

carbonate, and 0.07 M copper sulfate, 2.4 M NaOH, pH 10.6) were prepared, incubated for 15 min at room temperature, and the absorbance was measured at 330 nm using the Synergy H1.

References[11]

1. Kramer, G. J.; Mohd, A.; Schwager, S. L.; Masuyer, G.; Acharya, K. R.; Sturrock, E. D.; Bachmann, B. O., Interkingdom pharmacology of Angiotensin-I converting enzyme inhibitor phosphonates produced by actinomycetes. *ACS Med Chem Lett* **2014,** *5* (4), 346-51.

2. Dellafiora, L.; Dall'Asta, C.; Cozzini, P., Ergot alkaloids: From witchcraft till. *Toxicol Rep* **2015,** *2*, 535-545.

3. Baroni, M.; Cruciani, G.; Sciabola, S.; Perruccio, F.; Mason, J. S., A common reference framework for analyzing/comparing proteins and ligands. Fingerprints for Ligands and Proteins (FLAP): theory and application. *J Chem Inf Model* **2007,** *47* (2), 279-94.

4. Jones, G.; Willett, P.; Glen, R. C.; Leach, A. R.; Taylor, R., Development and validation of a genetic algorithm for flexible docking. *J Mol Biol* **1997,** *267* (3), 727-48.

5. Eugene Kellogg, G.; Abraham, D. J., Hydrophobicity: is LogP(o/w) more than the sum of its parts? *Eur J Med Chem* **2000,** *35* (7-8), 651-61.

6. Dellafiora, L.; Marchetti, M.; Spyrakis, F.; Orlandi, V.; Campanini, B.; Cruciani, G.; Cozzini, P.; Mozzarelli, A., Expanding the chemical space of human serine racemase inhibitors. *Bioorg Med Chem Lett* **2015,** *25* (19), 4297-303.

7. Marseglia, A.; Dellafiora, L.; Prandi, B.; Lolli, V.; Sforza, S.; Cozzini, P.; Tedeschi, T.; Galaverna, G.; Caligiani, A., Simulated Gastrointestinal Digestion of Cocoa: Detection of Resistant Peptides and In Silico/In Vitro Prediction of Their Ace Inhibitory Activity. *Nutrients* **2019,** *11* (5).

8. Dellafiora, L.; Paolella, S.; Dall'Asta, C.; Dossena, A.; Cozzini, P.; Galaverna, G., Hybrid in Silico/in Vitro Approach for the Identification of Angiotensin I Converting Enzyme Inhibitory

Peptides from Parma Dry-Cured Ham. *J Agric Food Chem* **2015,** *63* (28), 6366-75.
9. Akif, M.; Masuyer, G.; Schwager, S. L.; Bhuyan, B. J.; Mugesh, G.; Isaac, R. E.; Sturrock, E. D.; Acharya, K. R., Structural characterization of angiotensin I-converting enzyme in complex with a selenium analogue of captopril. *FEBS J* **2011,** *278* (19), 3644-50.
10. Dellafiora, L.; Galaverna, G.; Cruciani, G.; Dall'Asta, C., A computational study toward the "personalized" activity of alternariol - Does it matter for safe food at individual level? *Food Chem Toxicol* **2019,** *130*, 199-206.
11. Best, R. B.; Zhu, X.; Shim, J.; Lopes, P. E.; Mittal, J.; Feig, M.; Mackerell, A. D., Optimization of the additive CHARMM all-atom protein force field targeting improved sampling of the backbone φ, ψ and side-chain χ(1) and χ(2) dihedral angles. *J Chem Theory Comput* **2012,** *8* (9), 3257-3273.
